# Quantitative changes in mental health measures with 3MDR treatment for Canadian military members and veterans

**DOI:** 10.1002/brb3.2694

**Published:** 2022-07-18

**Authors:** Chelsea Jones, Lorraine Smith‐MacDonald, Matthew Robert Graham Brown, Ashley Pike, Eric Vermetten, Suzette Brémault‐Phillips

**Affiliations:** ^1^ Leiden University Medical Centre Leiden University Leiden the Netherlands; ^2^ Alberta Health Services, Edmonton Ottawa Canada; ^3^ Heroes in Mind, Advocacy and Research Consortium (HiMARC), Faculty of Rehabilitation Medicine University of Alberta Edmonton Canada; ^4^ Department of Computing Science University of Alberta Edmonton Canada; ^5^ ARQ National Psychotrauma Center Diemen the Netherlands; ^6^ Military Mental Health Service Dutch Ministry of Defence Utrecht the Netherlands; ^7^ Department of Occupational Therapy Faculty of Rehabilitation Medicine University of Alberta Edmonton Canada

**Keywords:** 3MDR, mental health, military, treatment‐resistant PTSD, veterans

## Abstract

**Objective:**

Military members and veterans are at elevated risk of treatment‐resistant posttraumatic stress disorder (TR‐PTSD) due to higher rates of exposure to potentially traumatic events during the course of duty. Knowledge of TR‐PTSD is limited, and specific protocols or evidence‐based TR‐PTSD therapies are lacking. Multimodal motion‐assisted memory desensitization and reconsolidation (3MDR) therapy is an emerging intervention for combat‐related TR‐PTSD. The purpose of this study was to preliminarily assess the effectiveness of 3MDR in addressing TR‐PTSD in Canadian military members and veterans.

**Methods:**

This study is a longitudinal mixed‐methods clinical trial. English‐speaking military members and veterans aged 18–60 with TR‐PTSD were recruited to participate. The intervention consisted of six sessions of 3MDR therapy. Quantitative data were collected pretreatment, posttreatment, and longitudinally at 1, 3, and 6 months after completion of 3MDR.

**Results:**

Results from the first 11 participants to complete the 3MDR protocol exhibited statistically significant improvement (surviving multiple comparison correction) in clinically administered and self‐reported scores for PTSD (CAPS‐5 and PCL‐5), moral injury (MISS‐M‐SF), depression (PHQ‐9), anxiety (GAD‐7), emotional regulation (DERS‐18), and resilience (CD‐RS‐25).

**Conclusion:**

The preliminary and exploratory results from this clinical trial support the growing body of literature illustrating 3MDR as an effective treatment for military‐related TR‐PTSD. These results are notable given participants' previous lack of success with frontline psychotherapeutic and pharmacological interventions. Given that there are currently very limited treatment options for TR‐PTSD, 3MDR could prove to be a valuable treatment option for military members and veterans with TR‐PTSD.

## Graphical abstract

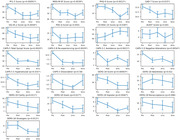

## 1 SIGNIFICANT OUTCOMES

• This study supports a growing body of literature illustrating that 3MDR may be an effective intervention for combat‐related treatment‐resistant PTSD in military members and veterans.
• Statistically significant improvements were found in all participant outcome scores related to PTSD, depression, anxiety, moral injury, emotional regulation, social support, and resilience.

## 2 LIMITATIONS

• The sample size utilized in this study is small, and caution should be used in generalizing the results presented in this study.
• Data were missing for some measures, for certain participants, at certain timepoints. It is possible that missing data may have affected the results, though we do not think this is likely.
• Control group data were unavailable at the time of analysis.
• Life events, circumstances, and the participant's level of support outside of the intervention varied and could not be fully controlled. The outcomes could have been influenced by factors additional to core intervention in question.

## 3 INTRODUCTION

Posttraumatic stress disorder (PTSD) is a condition that may develop following exposure to traumatic events that involve interpersonal violence, combat, life‐threatening accidents, or natural disasters (American Psychiatric Association, 2013; Yehuda et al., 2015). Military members are at elevated risk of PTSD due to higher rates of exposure to potentially traumatic events during the course of duty. A potentially traumatic event is one in which death, threatened death, or actual or threatened serious injury occurred. PTSD symptoms include negative cognitive intrusions, avoidance, hypervigilance, and alterations in mood, arousal, and reactivity (American Psychiatric Association, 2013; Yehuda et al., 2015). Among Canadian military members and veterans, PTSD rates have been reported at 5.3% (Zamorski & Boulos, 2014) and approximately 16% (Van et al., 2016) respectively, with PTSD rates increasing during the period in which Canadian Armed Forces members were involved in the war in Afghanistan (2001–2014) (Van et al., 2016). Rates of probable PTSD among UK military personnel has been reported to be 6.2% and 17.1% among veterans who had deployed in combat roles (Murphy et al., 2021; Stevelink et al., 2018). Among US and Dutch military members deployed during this global conflict, PTSD prevalence estimates reach up to 19% and 3%, respectively (Eekhout et al., 2016; Wagner & Jakupcak, 2012).

The severity of PTSD symptomatology may be worsened by cooccurring conditions that may also present with PTSD, as a result of the trauma exposure, of shared causal determinants or of the PTSD (Yehuda et al., 2015). Despite the prominent focus of the current literature on PTSD among military members, this population also demonstrates an increased risk for other mental health conditions. In the most recent survey of Canadian Armed Forces regular members, it was determined that the most prevalent mental health condition was alcohol abuse and dependence (31.9%), followed by major depressive disorder (15.7%) and then generalized anxiety disorder (12.1%) (Pearson et al., 2014). Similar trends have also been noted in Canadian veterans, with 21%–24% experiencing major depressive disorder and 18%–15% experiencing generalized anxiety disorder (Van et al., 2016). Military members and veterans who have served in post 9–11 conflicts have also been noted to exhibit elevated rates of behavioral challenging, including engagement in high‐risk lifestyles, inappropriate aggression, poor social and family functioning, and suicidal ideation (Bray et al., 2013; Pietrzak et al., 2010; Sayer et al., 2010; Taylor et al., 2009).

Gold standard treatments for military PTSD and associated mental illnesses include Cognitive Behavioral Therapy (CBT), Cognitive Processing Therapy (CPT), Prolonged Exposure (PE), and Eye‐Movement Desensitization and Reprocessing (EMDR) (Department of Defence & Veterans Affairs, 2017; Bisson et al., 2019). All of the above therapies are well‐evidenced and, in studies, have shown large effect sizes illustrating clinically significant reduction in symptoms (Watts et al., 2013). However, it is equally acknowledged that military members and veterans consistently have poorer clinical outcomes than their civilian counterparts in these treatments (Steenkamp et al., 2015; Forbes et al., 2019; Rehman et al., 2019). Although there is good evidence demonstrating the success of evidence‐based interventions at maintaining recovery at 6 to 12 months post intervention among civilian populations, the longevity of the recovery gains in military and veteran populations are less clear (Kline et al., 2018). To complement psychotherapeutic trauma treatments, pharmacological interventions (i.e., mood stabilizers, antiadrenergic agents/hypnotics, or atypical antipsychotic agents) have been suggested (Department of Defence & Veterans Affairs, 2017; Coventry et al., 2020). These treatments, however, may have undesired or untolerated side effects (Department of Defence & Veterans Affairs, 2017; Lee et al., 2016), and it is still unclear as to which pharmacological intervention is the most effective for military members experiencing PTSD (de Moraes Costa et al., 2020).

As a result, classification of treatment‐resistant PTSD (TR‐PTSD) has been adopted for the many military members and veterans who do not experience a clinically significant reduction in symptoms following receipt of at least two evidence‐based treatments (Hamblen et al., 2019; Forbes et al., 2019). As knowledge of TR‐PTSD is limited, general recommendations for TR‐PTSD have been suggested, but specific protocols or evidence‐based TR‐PTSD therapies are lacking, complicating clinical attempts to address or manage this condition (Hamner et al., 2004). A novel therapeutic known as multimodal motion‐assisted memory desensitization and reconsolidation (3MDR) therapy has been under investigation as a treatment option specifically targeting combat‐related TR‐PTSD.

### 3.1 Multimodal motion‐assisted memory desensitization and reconsolidation (3MDR)

Multimodal motion‐assisted memory desensitization and reconsolidation (3MDR) therapy is an emerging intervention for combat‐related TR‐PTSD. 3MDR is delivered in an immersive virtual reality system that combines a large visual display with a treadmill, allowing the patient to walk in the virtual environment. Many 3MDR studies have used the Computer‐Assisted Rehabilitation ENvironment (CAREN) or Gait Real‐time Interactive Laboratory (GRAIL) from Motek Medical BV, the Netherlands. The CAREN is a room‐sized, 3‐dimensional, virtual reality system with a centrally located treadmill that is surrounded by 240° floor‐to‐ceiling screens with motion‐capture technology. The multimodal nature of the intervention refers to the inclusion of (1) exposure to virtual reality (VR) visual imagery and auditory input, (2) walking, (3) a dual‐attention task, and (4) therapeutic context and relationship.

A 3MDR session typically lasts 90 min and includes three phases: (A) a *preplatform phase*, during which the patient chooses and orders symbolic representations in the form of pictures and selects music; (B) a *platform phase*, which involves a brief warm‐up during which the patient walks on the treadmill while listening to self‐selected music, followed by a series of seven 4‐ to 5‐min cycles of active therapy described below, and a cool‐down of walking to music; and (C) a *postplatform phase* providing an opportunity for review of the session and discussion of new insights and a self‐care plan. In each cycle of active therapy during the platform phase, the patient walks on the treadmill while viewing one of the previously selected images. The image gets larger as the patient approaches it in the virtual environment. A clinician stands alongside the treadmill and provides trauma counseling during this process. While walking and viewing the image, the patient also describes the traumatic scenario related to the image, as well as associated physical sensations, emotions, and thoughts. A subset of the patient's words is typed in by the therapist or an operator and is then displayed on the screen. A ball displaying a series of numbers is also presented on the screen for a period of 60 s, moving back and forth horizontally across the screen in the foreground of both the image and the emotion words. The patient must say the numbers as they appear on the moving ball. This process repeats with a total of seven cycles and seven different self‐selected images, during the platform phase. The theory and progression of 3MDR administration have been detailed in other publications (van Gelderen et al., 2018). The RCTs, and subsequent exploratory qualitative works, demonstrate preliminary effectiveness of 3MDR at reducing a number of PTSD, anxiety, and depressive symptoms in military members and veterans with TR‐PTSD at up to 26 weeks postintervention (van Gelderen et al., 2020a, 2020b; Bisson et al., 2020; Hamilton et al., 2021).

### 3.2 Aims of the study

The purpose of this study was to preliminarily assess the effectiveness of 3MDR in addressing TR‐PTSD in Canadian military members and veterans. We hypothesized that mental health measures (clinician and self‐reported standardized questionnaires) would improve with 3MDR treatment and this improvement would still be evident at the 6‐month follow‐up timepoint.

## 4 MATERIALS AND METHODS

### 4.1 Study design

A published protocol paper is available which fully describes the materials and methods of this mixed‐methods clinical trial (Jones et al., 2020). This paper presents an analysis of the pilot data from the first 11 participants who fully completed the study protocol as well as follow‐up sessions up to 6 months. This study was approved by the University of Alberta's Health Research Ethics Board (Pro00084466), and it received endorsement from the Canadian Armed Forces Surgeon General (E2019‐02‐250‐003‐0003).

### 4.2 Participants

A convenience sample of regular and reserve military members and veterans were recruited through established relationships with clinicians within the Canadian Armed Forces, Operational Stress Injury Clinics, the Royal Canadian Legion, and other local community service providers supporting military members and veterans. Participants were eligible for the study if they were English‐speaking, aged 18–60 years, possessed the capacity to walk on a treadmill for at least 45 min, and met the Diagnostic and Statistical Manual‐5 (DSM‐5) criteria for a diagnosis of PTSD, with symptoms lasting more than 3 months. To be eligible, participants also required a score of 30 or higher on the Clinician‐Administered PTSD Scale, Fifth Edition (CAPS‐5) interview, had trauma related to combat experiences, and were nonresponse to at least two types of evidence‐based PTSD treatments where at least one of these treatments was a psychotherapeutic intervention. It was permitted that the second treatment could be a pharmacological intervention. Participants were also required to be stable on their psychotropic medication for a period of 4 weeks before entering the trial. Participants with comorbidities were included if they satisfied the other criteria and PTSD was considered the primary diagnosis. Potential participants were also screened by a member of the research team to discuss their military employment/deployments, current and past medical history, history and experiences of previous PTSD interventions, and overall suitability prior to providing verbal and written consent to participate in the study.

### 4.3 Intervention

A full description of 3MDR as a therapeutic modality is available in other peer‐reviewed articles (Jones et al., 2020). A brief description is also provided in Section 3.1. The 3MDR intervention takes place in an immersive virtual reality (VR) environment. The current study used the CAREN VR system (Motek Medical BV, the Netherlands). As described in the Introduction, each session of 3MDR therapy includes three phases, *preplatform phase*, *platform phase*, and *postplatform phase*, with seven cycles of active therapy in the platform phase. A typical 3MDR session lasts 90 min, including the participant's walking on the platform for 45–60 min. For this study, the intervention consisted of six sessions of 3MDR: one session per week for six consecutive weeks. 3MDR was offered at a rehabilitation hospital in a large urban center in Western Canada.

### 4.4 Data collection

Standardized clinical outcome measures were filled out by patients via pencil and paper at the rehabilitation hospital. Data were collected before treatment commenced, after treatment was completed, and at follow‐up sessions 1, 3, and 6 months following the completion of treatment. The following clinician administered and self‐reported standardized quantitative measures were used.

• The PTSD Checklist for DSM‐5 (PCL‐5) (Weathers et al., 2018): The PCL‐5 is commonly utilized to measure PTSD symptom severity by self‐report. The PCL‐5 is a 17‐item questionnaire about symptoms in relation to an identified “stressful experience.” Each symptom can be rated on a 0–4 scale equaling from 0 to 80. This outcome measure is commonly utilized in research and clinical care to assess military members and veterans with PTSD and demonstrates strong reliability and validity.
• The Military Injury Symptom Scale—Military Short Form (MISS‐M‐SF) (Koenig et al., 2018): The MISS‐M‐SF was the primary outcome measure of interest for this study. The MISS‐M‐SF is a reliable and valid measure of MI symptoms that can be used to screen for MI and monitor response to treatment in veterans and active duty military with, or without, diagnosed PTSD (Koenig et al., 2019). The possible range of scores is from 10 to 100. The total score is an indication of functional impairment caused by MI.
• The DSM‐5 Clinician‐Administered PTSD Scale, Fifth Edition (CAPS‐5) (Blake et al., 1995): The CAPS‐5 is a 29‐item structured interview for assessing PTSD diagnostic status and symptom severity (Blake et al., 1995). The CAPS is the gold standard in PTSD assessment and can be used to make a current (past month) or lifetime diagnosis of PTSD or to assess symptoms over the past week. The CAPS‐5 was conducted focusing on the worse month and the last month to assess the appropriate baseline. We collected the Life Events Checklist for DSM‐5 (LEC‐5) during the baseline assessment.
• The Patient Health Questionnaire‐9 (PHQ‐9) (Kroenke et al., 2001): The PHQ‐9 is commonly used to measure the severity of depression. The PHQ‐9 incorporates DSM‐IV depression diagnostic criteria into a 9‐item, self‐report outcome measure. Responses represent the frequency of symptoms in the past two weeks and each symptom can be rated a 0–3 scale (Kroenke et al., 2001). A score between 5 and 9 indicates mild depression; 10 and 14 indicates moderate depression; 15 and 19 indicates moderately severe depression; and 20 and 27 indicates severe depression.
• The Generalized Anxiety Disorder Scale‐7 (GAD‐7) (Spitzer et al., 2006): The GAD‐7 is used to measure the severity of anxiety. This self‐report scale consists of 7 items, and responses represent the frequency of symptoms in the two past weeks and are given on a 0–3 scale (Spitzer et al., 2006). Scores between 5 and 9 indicate mild anxiety; 10 and 14 indicate moderate anxiety; and 15 and 21 indicate severe anxiety.
• The Peritraumatic Dissociation Event Questionnaires (PDE‐Q) (Birmes et al., 2003): The PDE‐Q is a 10‐item test that measures the extent of dissociation at the time of the traumatic event, and in the minutes and hours that followed. Studies suggest that dissociation increases the risk of developing PTSD (Birmes et al., 2003). PDEQ administration and scoring takes under 5 min each. All items are scored from 1 (not at all true) to 5 (extremely true), and the total score is the sum of all items (Birmes et al., 2003). A score above 15 is indicative of significant dissociation (Birmes et al., 2003).
• The Alcohol Use Disorder Identification Test (AUDIT) (Bradley et al., 2003): The AUDIT is an alcohol self‐report, 10‐item questionnaire that aims to help identify persons who are hazardous drinkers or have active alcohol use disorders. A score of 8 or more is considered to indicate hazardous or harmful alcohol use.
• The Difficulties in Emotion Regulation (DERS‐18) Scale (Victor & Klonsky, 2016): The DERS‐18 provides various measures of emotional regulation including the DERS‐18 overall score in the range of 18–90 and six subscores ranging from 3 to 18: Awareness, Clarity, Goals, Impulse, Awareness, and Strategies. Items are rated on a scale of 1 (“almost never [0%−10%]”) to 5 (“almost always [91%−100%]”). Higher scores indicate more difficulty in emotion regulation.
• The Outcomes Questionnaire‐45 (OQ‐45) (Lambert et al., 1996): The OQ‐45 is a self‐report inventory measuring social functioning. It consists of 45 items, which are rated on a 5‐point scale and reflect three domains: symptomatic distress, interpersonal relationships, and social role (Lambert et al., 1996). A total score of 63 or more indicates symptoms of clinical significance, and a difference of 14 points or more (between sessions) indicates a significant change in symptoms.
• The Connor Davidson Resilience Scale (CD‐RISC) (Connor & Davidson, 2003): The CD‐RISC is a tool utilized to measure perceived resilience within 17 domains. The tool consists of a 25‐item scale within these domains. This tool has been studied extensively and has been demonstrated to be valid and reliable when utilized with survivors of various traumas and PTSD (Connor & Davidson, 2003). We collected the CD‐RISC‐25 at timepoints pretreatment, posttreatment, or at 1‐, 3‐, or 6‐month follow‐up.

### 4.5 Statistical analysis

Participants had the option to not fill in all the questions in the self‐report questionnaires, as required by the University of Alberta's Health Research Ethics Board. Though most participants did answer all the questions, a small proportion of answers were missing. Mean imputation was used to fill in missing values. Specifically, for questionnaire scores where a given participant provided at least 75% of the answers required to generate the score, any missing answers were filled in with mean imputation. In cases where the participant provided less than 75% of the answers contributing to a questionnaire score, that questionnaire score was treated as missing. Considering all score values computed for all participants, questionnaires, and timepoints, 95.2% of all score values were computed from full data, 4.5% of score values were computed with 75% or more of the underlying answers available (using mean imputation for missing answers), and 0.3% of score values were treated as missing because less than 75% of the underlying answers were available. Therefore, in a very small proportion of questionnaire scores, some scores were missing for specific participants, for specific timepoints. In addition, the COVID‐19 pandemic created disruption in some of the data collection sessions, meaning that data were not collected for certain participants for certain timepoints. Handling of missing data is described below.

To examine changes in mental health scores over time, linear models and permutation testing were used. For a given score, the dependent values were the scores for each participant at various timepoints. The linear model included a linear term for change over time as well as one offset term per participant to capture between‐subject variability. The model was fit to the data using least squares. The fitted slope parameter from the linear term provided a measure of a score's change over time. Where necessary, the linear model was adjusted to handle missing data by removing the missing data points from the dependent score values and by removing the corresponding values in the linear model, before fitting the model to the data.

Permutation testing was used to test the estimated slope parameter for statistical significance, for each score. Permutation testing was chosen because it is a nonparametric method that does not require assumptions about the shape of the statistical distribution of the data (including no assumption that the data are normally distributed). For each score, we generated an empirical distribution of the estimated linear slope parameter including 100,0000 samples. A total of 99,999 iterations of permutation were used to generate all but one of the samples, and the actual estimated slope parameter comprised the last sample, as is standard practice. On each iteration, the timepoint labels were permuted randomly and separately within each participant, and the linear model was then fit to the permuted data to generate an estimated slope parameter. Where missing data points were present, permutation did not interact with the missing data. (That is, a missing data point was never exchanged with a valid data point during permutation.) The actual estimated slope parameter was then compared against the empirical distribution to generate a *p* value.

In total, permutation tests of linear changes were performed for 21 different scores. To address multiple comparisons, we used the Benjamini–Hochberg procedure for false‐discovery rate (FDR) correction (Benjamini & Hochberg, 1995).

## 5 RESULTS

### 5.1 Demographics

Table 1 presents demographics for the 11 participants analyzed in this study. All participants were Canadian active military members or veterans who had been deployed to one or more combat theatres during their careers.

**TABLE 1:** Demographics

Gender	Age	Marital status	Employment status	
Female, 1 (9%) Male, 10 (91%)	Mean: 45.4 ± 6.8 Range: 30.9–54.3 30–39, 2 (18%) 40–49, 6 (55%) 50–59, 3 (27%)	Common law, 2 (18%) Divorced, 1 (9%) Married, 5 (45%) Separated, 1 (9%) Single, 2 (18%)	No, 5 (45%) Yes, 6 (55%)	
Military status	Enrollment era	Rank	Element	Years of service
Active, 3 (27%) Veteran, 8 (73%)	1976–1990, 2 (18%) 1991–2000, 8 (73%) 2001–2015, 1 (9%)	Junior NCM, 6 (55%) Senior NCM, 4 (36%) Unknown, 1 (9%)	Air, 2 (18%) Land, 9 (82%)	5–10, 2 (18%) 11–15, 1 (9%) 20+, 8 (73%)

*Note*: Demographic characteristics are presented for 11 participants, including numbers and percentages (in brackets) of participants falling in the various categories for each characteristic. In the Age column, mean and standard deviation as well as range are also presented.

NCM = noncommissioned member.

### 5.2 Changes in mental health measures

**FIGURE 1:**
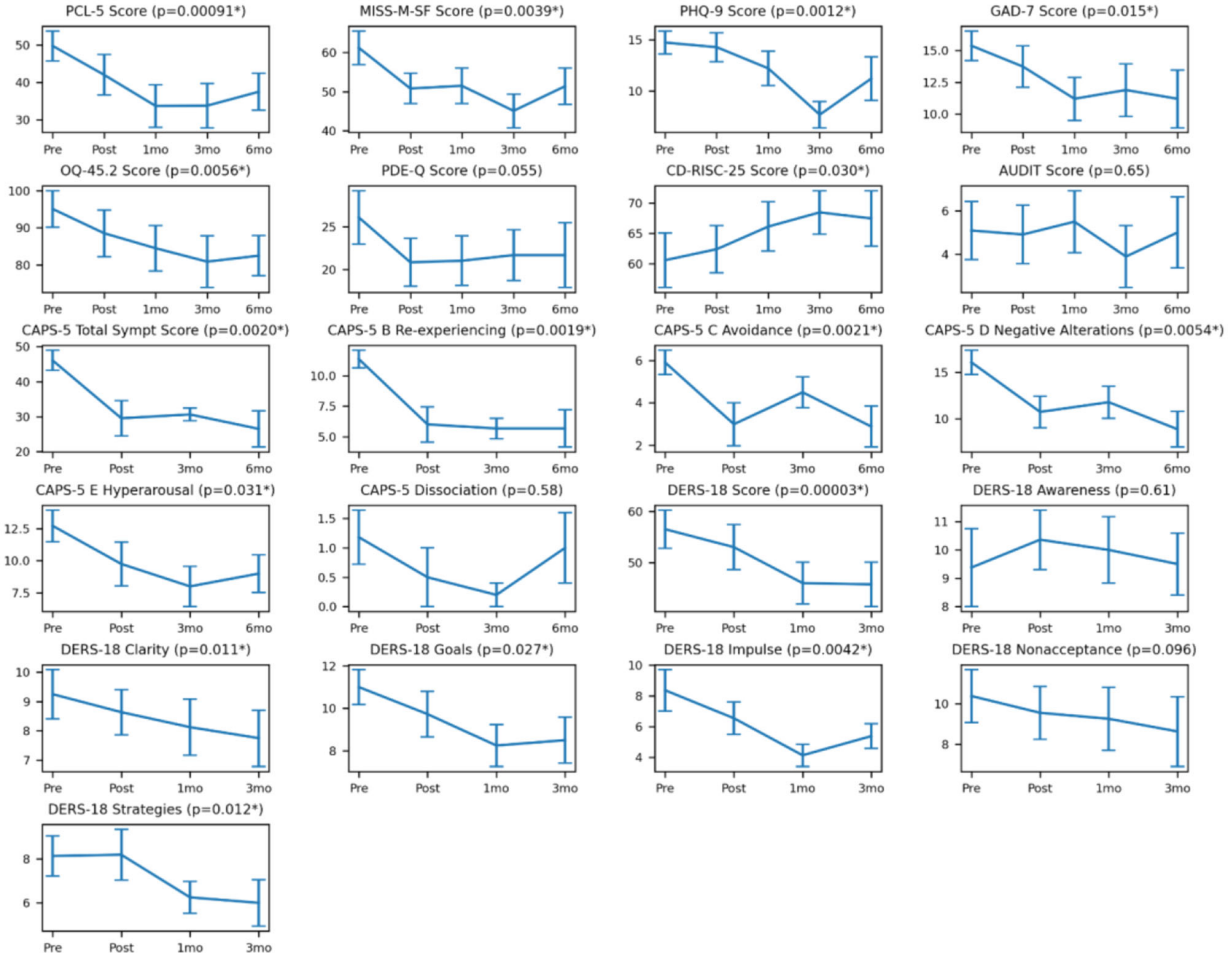
Changes in outcome scores from preintervention to 6 months postintervention. Data points show means across the 11 participants. Error bars denote standard error of the mean. Pre = pretreatment timepoint. Post = posttreatment timepoint. 1 mo, 3 mo, and 6 mo denote 1‐, 3‐, and 6‐month follow‐up timepoints. For some scores, data were not collected at every timepoint (see Section 4.4). *p* Values in brackets indicate significance on tests of linear change over time for each score. *p* Values were computed using permutation testing on the fitted slope parameter for the linear term of a linear model. *p Values with an asterisk survive FDR multiple comparison correction (threshold *p* = .031)

Mental health measures, including scores derived from self‐report questionnaires for the different timepoints, are shown in Table 2. Figure 1 shows changes over time for all scores. Multiple scores showed improvement from the pre‐ to post‐timepoint, with improvement remaining until the 6‐month follow‐up. It should be noted that the DERS‐18 was not measured at 6 months, and changes in DERS‐18 scores were studied only up to the 3‐month follow‐up. Scores exhibiting statistically significant improvement (surviving multiple comparison correction) included the PCL‐5 Score, MISS Score, PHQ‐9 Score, GAD‐7 Score, OQ‐45.2 Score, CD‐RISC‐25 Score, CAPS‐5 Total Symptom Score, CAPS‐5 B Re‐experiencing Score, CAPS‐5 C Avoidance Score, CAPS‐5 D Negative Alterations Score, CAPS‐5 E Hyperarousal Score, DERS‐18 Score, DERS‐18 Clarity Score, DERS‐18 Goals Score, DERS‐18 Impulse Score, and DERS‐18 Strategies Score. The PDE‐Q Score was close to showing significance (*p* = .055). The *p*‐value threshold for FDR multiple comparison correction was computed to be .031.

**TABLE 2:** Main results

Score	Pre	Post	1 Mo	3 Mo	6 Mo	Est slope	*p* Value
PCL‐5 Score	49.7 ± 12.6	42.0 ± 17.0	33.7 ± 17.1	33.8 ± 16.6	37.5 ± 14.7	–3.4	.00091*
MISS‐M‐SF Score	61.2 ± 12.9	50.8 ± 12.3	51.5 ± 13.6	45.1 ± 12.1	51.4 ± 14.0	–2.3	.0039*
PHQ‐9 Score	14.7 ± 3.6	14.3 ± 4.5	12.2 ± 5.1	7.7 ± 3.6	11.2 ± 6.4	–1.2	.0012*
GAD‐7 Score	15.4 ± 3.7	13.7 ± 5.1	11.2 ± 5.1	11.9 ± 5.8	11.2 ± 6.8	–1.0	.015*
OQ‐45.2 Score	95.1 ± 15.5	88.5 ± 19.9	84.5 ± 18.4	80.9 ± 19.7	82.5 ± 16.2	–2.8	.0056*
PDE‐Q Score	26.1 ± 9.9	20.8 ± 8.8	21.0 ± 8.7	21.7 ± 8.4	21.7 ± 10.7	–0.8	.055
CD‐RISC‐25 Score	60.5 ± 14.2	62.4 ± 12.4	66.1 ± 12.2	68.4 ± 10.1	67.4 ± 12.8	1.2	.030*
AUDIT Score	5.1 ± 4.3	4.9 ± 4.3	5.5 ± 4.3	3.9 ± 4.0	5.0 ± 4.9	–0.0	.65
CAPS‐5 Total Symptom Score	46.1 ± 9.1	29.5 ± 13.4	–	30.6 ± 3.6	26.4 ± 14.7	–6.7	.0020*
CAPS‐5 B Re‐experiencing	11.4 ± 2.3	6.0 ± 3.8	–	5.7 ± 1.9	5.7 ± 4.3	–1.8	.0019*
CAPS‐5 C Avoidance	5.9 ± 1.8	3.0 ± 2.7	–	4.5 ± 1.6	2.9 ± 2.7	–1.1	.0021*
CAPS‐5 D Negative Alterations	16.1 ± 4.1	10.8 ± 4.5	–	11.8 ± 3.4	8.9 ± 5.4	–2.4	.0054*
CAPS‐5 E Hyperarousal	12.7 ± 3.9	9.8 ± 4.5	–	8.0 ± 3.2	9.0 ± 4.2	–1.5	.031*
CAPS‐5 Dissociation	1.2 ± 1.5	0.5 ± 1.3	–	0.2 ± 0.4	1.0 ± 1.7	–0.1	.58
DERS‐18 Score	56.5 ± 9.9	53.0 ± 13.9	46.0 ± 10.8	45.8 ± 11.5	–	–3.8	.00003*
DERS‐18 Awareness	9.4 ± 3.7	10.4 ± 3.3	10.0 ± 3.1	9.5 ± 2.9	–	–0.1	.61
DERS‐18 Clarity	9.2 ± 2.2	8.6 ± 2.4	8.1 ± 2.5	7.8 ± 2.5	–	–0.5	.011*
DERS‐18 Goals	11.0 ± 2.2	9.7 ± 3.4	8.2 ± 2.6	8.5 ± 2.9	–	–0.9	.027*
DERS‐18 Impulse	8.4 ± 3.6	6.5 ± 3.4	4.1 ± 1.9	5.4 ± 2.1	–	–1.0	.0042*
DERS‐18 Nonacceptance	10.4 ± 3.5	9.5 ± 4.1	9.2 ± 4.1	8.6 ± 4.6	–	–0.6	.096
DERS‐18 Strategies	8.1 ± 2.4	8.2 ± 3.6	6.2 ± 1.9	6.0 ± 2.8	–	–0.7	.012*

*Note*: Time‐series analyses of questionnaire and structured interview scores from 11 participants. Pre, post, 1 mo, 3 mo, and 6 mo indicate mean ± standard deviation at timepoints pretreatment, posttreatment, or at 1‐, 3‐, or 6‐month follow‐up. Certain instruments were not collected at certain timepoints, as indicated by a dash. Est slope indicates the estimated slope from a linear model. *p* Values were computed with permutation testing of the estimated slope values. *p* Threshold for FDR multiple comparison correction was computed to be .031.

* *p* Values survive FDR multiple comparison correction.

## 6 DISCUSSION

The purpose of this study was to explore preliminary waitlist‐control trial data evaluating whether 3MDR was an effective trauma intervention for a small sample of Canadian military members and veterans with TR‐PTSD. The results demonstrated statistically significant changes in symptoms of PTSD, major depressive disorder, generalized anxiety disorder, emotional regulation, resilience, social functioning and close to significant changes for peritraumatic stress reactions. Despite the small sample size, these results are notable, considering that participants only received six 1‐h 3MDR sessions without the addition of adjunct pharmacological or psychotherapeutic treatments and considering that these changes were sustained for a period of at least 6 months.

The results support the growing body of evidence that 3MDR may be an effective treatment for combat‐related TR‐PTSD. Randomized control trials from the Netherlands and the United Kingdom have all shown a statistically significant reduction in CAPS‐5, PCL‐5, and GAD‐7 scores (van Gelderen et al., 2020a; Bisson et al., 2020). This preliminary evidence supports the hypothesis that 3MDR is effective in addressing the avoidance, anxiety, and fear‐based components associated with both PTSD and generalized anxiety disorder. While the specific mechanisms associated with overcoming these components within 3MDR remain unknown, a key factor may be the immersive virtual reality environment in which participants are safely exposed to traumatic memories and uncomfortable bodily sensations during 3MDR therapy. In van Gelderen et al.’s (2020b) and Hamilton et al.’s (2021) qualitative explorations of the perceived effective treatment processes of 3MDR, participants noted being unable to use personal avoidance techniques and having to face the most feared part of the traumatic memory. Such results align with emotional processing theory (Foa & Kozak, 1986), which holds that a reduction of symptoms requires a modification of the affective memory, enabling emotional processing such that the trauma‐related information no longer evokes fear (Foa & Kozak, 1986; Rauch & Foa, 2006). Other emerging theories postulate the importance of movement, such as walking, and its positive effect on divergent thinking, which may assist the 3MDR client in reframing previously held beliefs and schemas regarding guilt, shame, responsibility, grief, and anger.

The alignment between theory and results is much less clear for how 3MDR may impact emotions and mood. Results regarding major depression have been mixed. Jetly et al. (2017) and Bisson et al. (2020) did not find a statistical change in their participants' PHQ‐9 scores, and van Gelderen et al. (2020a) did not find a change with the Hospital Anxiety and Depression Scale (HADS). Conversely, the current study observed statistically significant changes in both the CAPS‐5 D Negative Alterations Score and the PHQ‐9, which raises an interesting question regarding the potential relationship between major depressive disorder, TR‐PTSD, and 3MDR. The DERS‐18 overall score and DERS‐18 subscale scores of clarity, goals, impulse, and strategies all showed statistically significant changes. However, the lack of significant changes in the DERS‐18 subscales of awareness and nonacceptance generates queries regarding how the underlying mechanism of emotional changes during 3MDR may work. A basic tenet of emotional regulation highlights that three central principles are awareness (monitoring), evaluating and if necessary, controlling or modifying behaviors (Foa & Kozak, 1986).

The current results tentatively support the theory that, in PTSD, emotional undermodulation (i.e., hypervigilance, heightened experiences of negative emotions, and poor inhibition and behavioral adaptations in response to negative emotions) may be an important aspect of PTSD (Yehuda et al., 2015). Difficulties with emotional regulation are highly correlated with severity and maintenance of combat‐related PTSD (Raudales et al., 2020; Spies et al., 2020). Aspects of emotional dysregulation such as maladaptive or absent regulatory strategies, avoidance and repression of emotions, and/or unawareness of emotions have been linked to PTSD symptom severity (Christ et al., 2021). The use of avoidance and repression of emotions strategies has been noted to be particularly frequent and problematic within military and veteran populations who have PTSD (Litz, 1992). Veterans who experienced emotional numbing and anhedonia have also been found to report more PTSD symptoms (Kashdan et al., 2007). It may be that, as military members and veterans engage in 3MDR, the immersive components addressing the traumatic avoidance may also be inadvertently addressing the emotional avoidance, hypervigilance, and poor inhibition strategies. On the other hand, previous 3MDR studies have not shown any significant change in AUDIT scores, which is noteworthy as substance misuse has commonly been seen as a maladaptive strategy to avoid emotional pain (van Gelderen et al., 2020a; Bisson et al., 2020; Jacobsen et al., 2001).

The role of emotional regulation may also help to explain the statistical changes in the MISS and CD‐RISC‐25 scores. Research into moral injury (MI) has predominantly focused on the resulting problematic emotions (e.g., guilt, shame, anger, bitterness, hatred, contempt, and disgust), self‐appraisal, and social isolation (Frankfurt & Frazier, 2016; Griffin et al., 2019). Farnsworth et al. (2014) have proposed that one functional approach to understand MI is to observe the role of moral emotions in its development. Vermetten and Jetly (2018) have also argued for the role of guilt and shame as drivers for chronicity of PTSD. Interestingly, MI has been strongly associated with depressive symptoms (Currier et al., 2017; Koenig et al., 2019; Currier et al., 2015). Authors of qualitative evaluations have also suggested that morally injurious outcomes and depressive psychopathology cooccur (McCormack & Ell, 2017; Purcell et al., 2016; Williamson et al., 2019). Our results are consistent with this as both the MISS and the PHQ‐9 scores changed post 3MDR. Little empirical research, however, has addressed this possible relationship. Williamson et al.’s (2019) qualitative research noted that UK veterans suffering from MI often experienced negative emotions and cognitive patterns in addition to emotional numbing. Similarly, Protopopescu et al. (2021) suggest that the severity of MI is positively correlated with the severity of cluster C and D PTSD symptoms, though interestingly ER difficulties were not correlated with MI. It is also unclear if changes in the OQ‐45 were because of changes in PTSD or MI symptoms as both conditions have been associated with negative social impacts (Tsai et al., 2012; Chesnut et al., 2020).

Emotional regulation may also play an underappreciated, and yet critical role in resilience. Bion (1961) argued that understanding emotions, managing emotions, and maintaining one's relationships are critical in the process of fostering resilience. Barton and William (2019) argued that understanding the underlying emotions in oneself can lead to more information about interpersonal and group‐level dynamics, which gives people more agency and self‐efficacy, leading to individual and group level resilience. Schneider et al. (2013) also found that emotional intelligence related to lower threat appraisals, more modest declines in positive affect, less negative affect, and challenged physiological responses to stress. Likewise, Magnano et al. (2016) showed that emotional intelligence plays a significant role in resilience. In the same vein, Armstrong et al. (2011) revealed that emotional intelligence was related to psychological resilience. Finally, Liu and Boyatzis (2021) also illustrated that people with a high level of emotional intelligence showed a greater degree of resilience with the dimensions of emotional repair being the most significant in the emotional intelligence dimensions. Again, further research will be needed to determine how and why 3MDR treatment supported an increase in participants' resilience.

### 6.1 Limitations

This study has a number of limitations. The sample size for this pilot analysis was small (*n* = 11) largely due to restrictions related to the COVID‐19 pandemic. Control data are also currently missing as the number of control group participants with complete data was smaller than the intervention group at the time of COVID‐19 restrictions. This would not allow for an accurate and meaningful comparison at this time results from the full waitlist control clinical trial will be forthcoming. Caution regarding possible overgeneralization is highly warranted. Second, it is possible that missing data (4.5% of score values were computed using mean imputation for missing answers, and 0.3% of score values were treated as missing because less than 75% of the underlying answers were available) within the outcome measures may have impacted results. Third, although additional trauma interventions were controlled for, other life events, circumstances, and the participant's level of support outside of the 3MDR intervention varied and could not be fully controlled. The outcomes described here could have been influenced by factors additional to 3MDR therapy that were not captured in our analysis.

## 7 CONCLUSIONS

The exploratory results from this trial support the hypothesis that 3MDR is an effective treatment for combat related TR‐PTSD. The results from the first 11 participants of this study show promise; analysis of this preliminary data demonstrated statistically significant decreases in symptoms of PTSD, generalized anxiety disorder, major depressive disorder, and MI after 3MDR and at 6 months posttreatment. Additionally, participants demonstrated an increase in emotional regulation, resilience, and social functioning. These results are notable given participants' previous lack of success with frontline psychotherapeutic and pharmacological interventions. Given there are currently limited treatment options for TR‐PTSD, 3MDR may prove to be a valuable treatment option for military members and veterans with TR‐PTSD. Further theory and research are needed to address the current lack of understanding of the mechanisms underlying 3MDR's apparent effectiveness.

## CONFLICT OF INTEREST

Professor Vermetten created 3MDR but would not stand to benefit financially were it to be adopted into routine clinical practice. The other authors declare no conflicts of interest.

### PEER REVIEW

The peer review history for this article is available at https://publons.com/publon/10.1002/brb3.2694

## Data Availability

Due to privacy/ethical restrictions, the data used in this study are available only on request from the corresponding author, CJ or SBP. The data are not publicly available due to their containing information that could compromise the privacy of research participants.
